# Fully Self-Supervised Out-of-Domain Few-Shot Learning with Masked Autoencoders

**DOI:** 10.3390/jimaging10010023

**Published:** 2024-01-16

**Authors:** Reece Walsh, Islam Osman, Omar Abdelaziz, Mohamed S. Shehata

**Affiliations:** Irving K. Barber Faculty of Science, University of British Columbia, Kelowna, BC V1V 1V7, Canada; reece.walsh@ubc.ca (R.W.); islam.osman@ubc.ca (I.O.); mohamed.sami.shehata@ubc.ca (M.S.S.)

**Keywords:** few-shot learning, self-supervised, image classification, out-of-domain

## Abstract

Few-shot learning aims to identify unseen classes with limited labelled data. Recent few-shot learning techniques have shown success in generalizing to unseen classes; however, the performance of these techniques has also been shown to degrade when tested on an out-of-domain setting. Previous work, additionally, has also demonstrated increasing reliance on supervised finetuning in an off-line or online capacity. This paper proposes a novel, fully self-supervised few-shot learning technique (FSS) that utilizes a vision transformer and masked autoencoder. The proposed technique can generalize to out-of-domain classes by finetuning the model in a fully self-supervised method for each episode. We evaluate the proposed technique using three datasets (all out-of-domain). As such, our results show that FSS has an accuracy gain of 1.05% , 0.12% , and 1.28% on the ISIC, EuroSat, and BCCD datasets, respectively, without the use of supervised training.

## 1. Introduction

Few-shot learning (FSL) has allowed deep learning models to learn from datasets with limited labels. FSL has recently attracted massive attention as it provides a solution for image classification of datasets with limited labels (e.g., medical images). Many approaches have been introduced to address the problem of learning with limited data. These approaches are categorized into three techniques: (1) metric-based, (2) optimization-based, and (3) self-supervised-based.

In metric-based techniques, the learning model learns a distance metric to distinguish between different classes [1,2,3]. An example of metric-based techniques is Prototypical Networks (ProtoNet) [4]. ProtoNet is widely used to embed the input images into a high-level representation, which can be used to classify the images easily.

In prior optimization-based techniques, the models are trained to be task-agnostic and are easily adapted to new tasks [5,6,7]. An early attempt at optimization-based techniques is model-agnostic meta-learning (MAML) [8]. MAML aims to learn a set of parameters that can be used as a good initialization for any new task. Hence, the model can adapt its parameters to a new task with a few labels and a few training iterations.

More recently, self-supervised learning (SSL) has been introduced as a solution to FSL. SSL leverages unlabeled data to learn useful knowledge that can be transferred to solve new tasks with limited labels [2,9,10]. The first attempt at using SSL in FSL is AmdimNet [9]. In AmdimNet, during the pretext phase, the model is trained to maximize the mutual information of two views on the input image. In the downstream phase, the model embeds the query set, and classification is performed based on the distance between the query embedding and the class centroid.

Although these models have achieved adequate performance in FSL, they still face two main problems: (1) when these models are tested on out-of-domain samples, their performance degrades tremendously, and (2) these models demonstrate increasing reliance on supervised finetuning through the use of the support set (the labelled data) to classify the query set.

To this end, we propose a fully self-supervised few-shot learning technique (FSS) that utilizes a masked autoencoder. Our contributions are two-fold:

- We introduce masked image modelling through a masked autoencoder into the few-shot learning paradigm without the need for labelled data for finetuning.
- We conduct experiments which show that a model can generalize to out-of-domain samples and classify the query set without needing labelled data to finetune.

To the best of our knowledge, this is the first attempt in self-supervised few-shot learning that does not use any labelled data. In our proposed model, an on-line self-supervised finetuning session is performed for each episode to adapt the model. Then, the finetuned model is used to classify the query set using a prototype-based classification.

## 2. Related Work

In this section, we review related literature. We provide a summary of similar and related techniques to our method. In particular, we investigate few-shot learning-based classification, meta-learning, metric learning, generative models, and self-supervised learning.

### 2.1. Few-Shot Learning-Based Classification

In few-shot image classification, a model is required to correctly classify a set of unlabelled images given a certain number of labelled images. The most common way to address this setting is episodic learning [11], in which data is drawn in batches from a dataset. The unique feature of episodic learning is that each batch has a defined number of classes and a defined set of labelled and unlabelled examples. To create an episodic batch $B_{E}=\left\{S,Q\right\}$ , first, a set of labels *L* is sampled from the data distribution over all possible subsets of labels. After that, images from the same distribution are sampled such that it has the same labels in *L*. Now, we can divide the images between *S* and *Q* such that $S=\{\left(s_{1},y_{1}\right),\ldots ,\left(s_{n},y_{n}\right)\}$ and $Q=\{\left(q_{1},y_{1}\right),\ldots ,\left(q_{m},y_{m}\right)\}$ , where *S* is the support set, *Q* is the query set, *n* is the number of examples in *S* (also called the shots), and *m* is the number of examples in *Q*. Also, w=|L| is defined to be the number of classes in the problem (also called the ’ways’). For further theoretical and experimental investigation of episodic learning, readers are encouraged to refer to [12].

#### 2.1.1. Meta-Learning

Meta-learning is concerned with a parameter-level approach to few-shot learning [13]. Typically, two networks are defined in this setup; one is the base and the other is the derived network. During an episode of training, the derived episode-specific network is trained on the labelled data (also called the ’support set’) of the episode. After fitting the derived network to the labelled examples, predictions are made for the unlabelled examples. These predictions are used to update the base network, thus gaining generalizability towards unseen examples. Following [14], meta-learning approaches can be divided into three categories: meta-representation, meta-optimizer, and meta-objective.

#### 2.1.2. Meta-Representation

Meta-representation is the category concerned with the learning methodology aspects that should be learned. When learning the parameters that control the learning process, some of these parameters can be learnable and others fixed. For this setting, parameters initialization, the optimizer, hyperparameters, and many more aspects related to the training could be determined by optimization on their level. MAML [8,15] is an example of parameter initialization meta-representation. MAML aims to learn model parameters to help the model quickly adapt to unseen tasks. The main idea is that some internal representations are more general than others, making it straightforward to find two sets of parameters, one that is robust to changes in all tasks and another that is sensitive to changes in each task. To this end, MAML involves a gradient of a gradient to achieve the aforementioned goal. For the other setting of optimizer meta-representation, it is the optimizer parameters that are the targets of the optimization process. While some works have addressed this setting by proposing networks that can learn some parameters of certain predefined optimizers [16,17], others have proposed that one optimizer step can be learned without having to adhere to a specific inner optimizer [13]. Overall, meta-representation is advantageous when choosing good parameters representing the problem without overcomplication. However, it is sensitive to meta-representation choice. In this work, we avoid the meta-representation scheme to reduce model complexity.

#### 2.1.3. Meta-Optimizer

Meta-optimization is concerned with the outer optimization strategy, in other words, the optimizer that optimizes other inner optimizers. The main family of meta-optimizer meta-learning is the gradient-based one. In this manner, outer gradient steps are typically a chain rule derivative of the inner ones that have actual model parameters. Works such as [18,19] calculate the gradient descent of the meta-objective with respect to the inner parameters via a chain rule. In this work, we do not require outer optimization steps. Instead, for each episode, the model is required to understand the underlying structure of the batch by masking out randomly chosen portions of each image and prompting the model to complete the missing portions. To this end, each inner step of the episode is not jointly optimized with other steps using a meta-optimizer. In conclusion, a meta-optimizer is efficient when it comes to adaptability to a new parameter space. However, the gradient-based approach might lead to sub-optimal results in a new task domain due to potential overfitting. Our method infers the required properties to be optimized from each new data distribution, resulting in a data-driven understanding.

#### 2.1.4. Meta-Objective

Meta-objective is the aspect associated with the objective of the outer optimization loop. In this work, we are concerned with the design of the episodes and the training paradigm. For the episode design, our goal is to adapt to a few shots, as in [8], rather than many shots [18]. Also, we calculate the validation loss one time at the end of each episode, which tends to result in better base model performance rather than fast adaptation [20]. Our model adopts online training, as in [21,22], where we can get the model to exploit the underlying structure of the data without using any labels.

#### 2.1.5. Metric Learning

Metric learning reformulates the image classification problem as a clustering problem by applying a distance measure to compare the sample similarity. Generally, a network is used to learn vector representations for images in the episode. These vector representations (also known as ’embeddings’) contain image features that are useful for robust image classification. Depending on these features and a distance metric, the model can classify a newly given query set using a support set. The most commonly used approach in the literature is Prototypical Networks where support classes centroids are used to classify query examples by choosing the nearest centroid class to a given query image.

### 2.2. Generative Models

Progress continues to be made in the development of generative techniques, particularly in terms of their ability to create high-quality output. Some noteworthy research directions in this field include variational autoencoders (VAEs) [23], generative adversarial networks (GANs) [24], and diffusion models [25]. The training process for these generative techniques typically involves methods like autoencoding input data, denoising input data, or utilizing a critic-based zero-sum loss to help the model learn from a training dataset. When generating new examples using a trained generative model, it usually involves using random input data sampled from a latent space. Depending on the specific training distribution, the input data can be conditioned to emphasize certain features or explore the commonalities [26] in the latent space.

In previous few-shot learning (FSL) techniques, VAEs [27], GANs [28], and diffusion models have demonstrated the potential to enhance few-shot classification accuracy. However, most of these techniques have primarily focused on image generation as their main application, which can be particularly challenging due to the vast range of potential image distributions. Recent research has explored embedding-based generative models, but they have had limited success, especially when tested with data from different domains. In contrast to these previous approaches, our method does not require specific data related to the input modality or critic-based loss functions for effective training.

An alternative approach was introduced by [29], which involves distribution calibration to address the challenge of learning from a limited number of samples. This method transfers statistics from classes with sufficient examples to those with few samples, thereby adjusting the distribution to allow for the generation of expanded inputs. However, this technique assumes that every dimension in the feature representation follows a Gaussian distribution, which may not always be the case, limiting its applicability in certain situations.

Masked image modelling (MIM), which is the core of this work, is considered a generative technique. It generates unseen portions of an image based on some other given portions exploiting the spatial correlation in the image.

### 2.3. Self-Supervised Learning

In recent years, self-supervised methods have become increasingly popular, particularly in the realm of natural language processing (NLP). Prominent examples include extensive language models like GPT [30,31,32] and BERT [33], which utilize self-supervised, masked pretraining to attain top-tier performance on NLP datasets.

In computer vision, self-supervised learning is a strategy in the field of representation learning, where a model is tasked with learning from unlabelled data. This approach can be better explained by looking at it through the lens of energy-based models (EBMs) [34]. In the context of self-supervised learning, the primary goal is to assign higher energy levels to inputs that are dissimilar in semantics, while assigning lower energy levels to semantically similar inputs. The current landscape of self-supervised learning encompasses both generative and non-generative methods, all of which align with this theoretical foundation.

Following [35], self-supervised learning techniques fall into four categories: deep metric, canonical correlation, self-distillation, and masked image modelling.

#### 2.3.1. Deep Metric

The deep metric category enhances a model’s semantic understanding by training it explicitly to deduce relationships between pixels in the input image. Concretely, the goal of the training is to push the embeddings of two inputs of the same class to be similar using contrastive loss [36]. Some works also encourage pushing away the embeddings of two inputs of different classes simultaneously while minimizing the distance between two inputs of similar class [37]. To ensure that two inputs are of the same class while not having access to labels in the pretaining (pretext) phase, the inputs are taken of the same image. Usually, the two inputs are two views of the same image. These views are obtained using a predefined set of view augmentations which preserves the semantics of the original image while introducing two images of different visual representations. The view augmentations often come from a predefined set of heuristically chosen combinations of image transformations, such as random resizing, colour jittering, random blurring, and random cropping. Despite empirically proven high image classification accuracy, the fixed set of augmentations may limit the model understanding to the cases present in the set. Tackling this, we do not require any additional augmentations, thus enabling the model to fully use the knowledge gained from completing masked portions of the input image.

#### 2.3.2. Canonical Correlation

Similar to Section 2.3.1, two augmented views of the same image are fed to two networks to extract two embedding vectors. The training paradigm aims to force the two vectors to have maximum cross-correlation while each one of them is required to have zero mean and identity covariance. Many works have tackled this setting, as in [38,39]. One closely related idea to our work is the Barlow Twins method [40]. Originally, it was introduced as a loss function to maximize the correlation between two embedding vectors. For a setting similar to ours [41], Barlow Twins was found to be empirically less effective than the regular mean squared error (MSE). In this work, we do not force the two representations to be maximally correlated. However, we depend on the masking strategy to internally extract useful correlated features.

#### 2.3.3. Self-Distillation

Following the same paradigm in Section 2.3.1 and Section 2.3.2, self-distillation involves two different augmented views of the same image where the two transformations are sampled from a set of hand-crafted image transformations. Moreover, the two transformations are fed to two networks, often called the student and the teacher. The ultimate goal is to make the embedding output of both networks as close as possible for two views of the same image, usually by means of the MSE loss. Notably, this category suffers from representation collapse, which happens when the two networks map the different views to the same zero or trivial vector that does not help generalize to unseen examples. Addressing this, the literature often describes freezing of the teacher network [42] and the adding of a small network on top of the student called the predictor. The teacher network weights are then updated using an exponential moving average of the student weights, as in [42,43]. In our work, we avoid any assumptions on a pretrained teacher model and only train our model on the given few-shot data, hence, reducing computational complexity.

#### 2.3.4. Masked Image Modelling

Closely related to our work and different from the approaches described in the previous sections, masked image modelling (MIM) addresses self-supervised learning by masking out portions of one input image and trying to reconstruct the hidden portions. In a masked autoencoder (MAE) [44], which inspires our work, an image is divided into a sequence of non-overlapping patches. A uniform random sampling strategy is applied to the patches to select 25% of them. The selected patches are then fed into a vision transformer (ViT) encoder to extract their feature embeddings. A lightweight decoder is used to decode the patches from the embedding space into the image space again. The decoder is prompted by aligning the latent embeddings and other mask tokens to take the place of the masked-out portions.

## 3. Materials and Methods

We propose fully self-supervised masked autoencoders for out-of-domain few-shot learning (FSS), a novel technique that adapts a vision transformer (ViT) [45] to new domains through the application of an on-line self-supervised finetuning session. Given an unlabelled episodic batch, FSS iteratively learns to reconstruct the randomly masked portions of the contents, thereby encouraging a holistic understanding of the distribution without the need for labels. During testing, we treat the ViT encoder as a metric backbone, enabling support prototype-based classification of the query set embeddings output by the FSS’s encoder. For the following sections, we explore FSS’s architectural composition, encoding process, and on-line finetuning in more detail.

### 3.1. Architectural Composition

**Figure 1:**
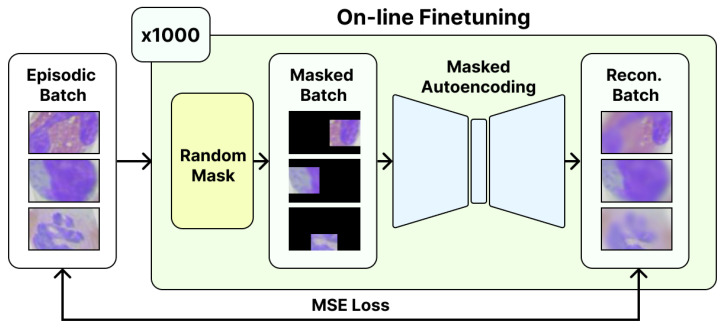
**Our proposed approach for FSS.** During testing, we perform an on-line finetuning session using a given episodic batch. The reconstruction loss is communicated as the mean squared error between the reconstructed episodic batch and the original episodic batch. No labels are used during this process.

FSS consists of a ViT-based encoder–decoder architecture that randomly masks and attempts to reconstruct a portion of patches within a given input image (illustrated in Figure 1). To this end, we base our implementation on previous work put forth by He et al. [44] and, thus, leverage several previously explored properties in our proposed approach.

First, we adopt the ViT-Large architecture as a basis for the encoder portion of FSS. We find that an ImageNet-1k pretrained instance of ViT-Large serves as an optimal starting point for reconstruction loss when finetuning on a new domain. Second, we include an asymmetric encoder–decoder architecture in FSS, as employed by He et al. [44] previously. Third and finally, we mask 75% of all patches in a given input image.

### 3.2. Masked Autoencoding

The reconstruction of masked data within a given image forms the basis for the self-supervised finetuning performed by FSS, a process also known as ’masked autoencoding’. This encoding and decoding process begins with our ViT encoder taking in an episodic batch of images as input. A selection of patches within each image is randomly obscured before each unmasked patch is embedded and used to provide context during reconstruction by the decoder. The network is finetuned in this process, learning to accurately reconstruct images through a new understanding of the underlying distribution. The loss function for a single image at this phase can be described as:

(1) $$MSE=\frac{1}{W*H}*\sum_{i=1}^{W}\sum_{j=1}^{H}\left|\right|{\hat{s}}_{ij}-s_{ij}{\left|\right|}_{2}^{2},$$

where *W* is the width of the input image, *H* is the height, ${\hat{s}}_{ij}$ is the predicted image pixel (predicted hidden portions are aligned with unhidden portions) at i,j , and $s_{ij}$ is the input image pixel at i,j . We find that reconstruction performance is heavily correlated with encoder accuracy when testing as a prototypical network, indicating that holistic understanding of a new domain can be gained through self-supervised reconstruction.

### 3.3. On-Line Finetuning

As one of the main contributions of our proposed approach, we observe that self-supervised finetuning with masked autoencoding can be conducted on a per-episode basis for an effective boost in out-of-domain few-shot accuracy. To this end, our model is finetuned in an on-line manner across each episode. During online finetuning, our model takes in an episodic batch and iteratively learns to accurately reconstruct randomly masked patches for each input image. For instance, given an episodic batch comprising $n_{q}$ query images (unlabelled) and $n_{s}$ support images (labelled), we loop for *N* iterations in the finetuning loop. In each finetuning loop iteration, we loop through each image in $n_{q}$ images and mask out a random portion of the image, then we prompt the model to fill in the masked parts. Subsequently, the model builds a deeper understanding of the domain to be able to fill in the masked portions; hence, the out-of-domain performance is enhanced. We specifically note that randomly resized cropping is key to preventing model overfit during finetuning.

### 3.4. Testing

We follow the testing approach by [4]. In this metric-learning approach, first, the pretrained model is used to compute the centroids of the support classes. After that, the embeddings of the query images are also calculated. To classify query images, each image embedding is compared to the support centroids. The class whose centroid is the nearest to the query image embedding is considered its class. Following that, in this phase, we have the labelled support set from which we compute the support centroids and the unlabelled query set. After finetuning on an episode has finished, the support set centroids are used to test the model on the query set to observe performance on the new domain. We find that a combination of the ViT-Large encoder and FSS’s online finetuning process provides a significant boost in performance across all the tested domains.

## 4. Results

In this section, we share few-shot learning classification accuracy results on EuroSAT [46], ISIC2018 [47], and BCCD WBC [48]. Additionally, we ablate several hyperparameters and properties of FSS in an out-of-domain setting. Using precedent from past approaches [11], all the data are samples from the respective datasets in an episodic format.

### 4.1. Datasets

As previously proposed by Vinyals et al. [11], we sample data from all the testing datasets in an episodic testing framework. Specifically, we test on three out-of-domain datasets: EuroSAT [46] (out-of-domain), satellite imagery from Europe with 10 testing classes, ISIC2018 [47] (out-of-domain), a dataset containing imagery of skin lesions with seven, and BCCD WBC [48] (out-of-domain), a dataset comprising white blood cell images with five classes.

#### 4.1.1. EuroSat

EuroSat is a Sentinel-2 satellite-based image dataset that contains 27,000 labelled and geo-referenced images. The dataset contains 10 classes with approximately 2000 to 3000 images per class. Each image has dimensions of 64 × 64 pixels. These images were taken from 34 European countries. Moreover, it is free and open source for all use cases (commercial and non-commercial).

#### 4.1.2. ISIC

The International Skin Imaging Collaboration (ISIC) dataset was introduced in a challenge that the ISIC organization held. The dataset consists of 12,000 images distributed across three different tasks: legion segmentation, attribute detection, and disease classification. Of interest is the disease classification dataset, which contains 10,015 training images, 193 validation images, and 1512 test images. The test images are comprised of 1196 images that are from the same source as the training images and 316 images from outside sources.

#### 4.1.3. BCCD

The Blood Cell Classification and Detection (BCCD) dataset has three main original classes: red blood cell (RBC), white blood cell (WBC), and platelet. The class that is heavily used in testing the out-of-domain performance of deep networks is the WBC class, with 372 images of five subclasses. Each image in the dataset has dimensions of 640 × 480 pixels.

### 4.2. Implementation

Taking inspiration from previous work in this domain, we base our approach on Hu et al.’s ViT-Large masked autoencoder model (24 layer encoder, 16 heads, 8 layer decoder, and a 16 × 16 px patch size). We additionally make use of a pretrained instance of this model for finetuning and testing. Code for this work can be found at: https://github.com/Brikwerk/FSS, accessed on 1 October 2023.

### 4.3. Experimental Setup

We share the hyperparameters of the conducted experiments in Table 1 for reproducibility.

### 4.4. Results

We evaluate the effectiveness of FSS across three image classification datasets while also comparing against other state-of-the-art few-shot learning approaches. Test results from our experiments are reported after on-line finetuning has been applied to the ViT-Large masked autoencoder for each episode. The few-shot test results are reported through use of the ViT-Large encoder as a prototypical network [4].

We follow the general paradigm of testing found in the few-shot literature [4,49]. Particularly, given an episodic batch $B_{E}=\left\{S,Q\right\}$ , first, a set of labels *L* is sampled from the data distribution over all possible subsets of labels. After that, images from the same distribution are sampled with the same labels in *L*. Now, we can divide the images between *S* and *Q* such that $S=\{\left(s_{1},y_{1}\right),\ldots ,\left(s_{n},y_{n}\right)\}$ and $Q=\{\left(q_{1},y_{1}\right),\ldots ,\left(q_{m},y_{m}\right)\}$ , where *S* is the support set and *Q* is the query set. Having created an episode, the accuracy is then evaluated for it. For more robust and realistic results, the model is tested on many episodes and the average accuracy is reported. The typical number of episodes is from 600 to 1000.

Table 2 and Table 3 both establish the effectiveness of FSS across all the tested out-of-domain datasets in a 5-way 5-shot setting. We observe that a fully self-supervised ViT-Large instance is competitive with other, fully supervised few-shot learning approaches. Furthermore, our experiments show that FSS is capable of achieving state-of-the-art results on out-of-domain datasets. We note, however, that the results from an in-domain perspective show reduced improvement relative to out-of-domain settings due to the lack of a need for self-supervised finetuning. This is due to the pretrained ViT-Large model we employ during testing leaving little room for reconstructive improvement.

In addition, we particularly highlight that the outcomes achieved through FSS are the result of label-less self-supervised learning. Unlike other supervised few-shot learning approaches, FSS can successfully adapt to out-of-domain settings through use of masked autoencoding. Our experiments indicate that effective self-supervised finetuning sessions can be conducted in an on-line manner using each respective episode. We note, however, that regularization of a given episode is necessary to combat overfitting by FSS.

Furthermore, we test the effectiveness of our online finetuning method by conducting experiments on ViT, where our online finetuning method shows greater performance than direct few-shot training, as depicted in Table 2 and Table 3.

**Figure 2:**
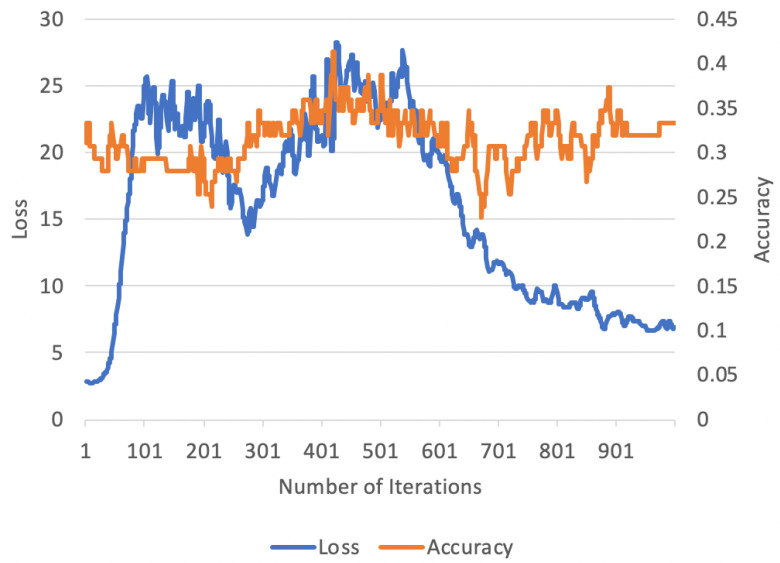
**Loss and accuracy for an ISIC episodic batch over 1000 finetuning iterations.** Notably, the loss spikes before converging at a new minimum. Although the accuracy, on the other hand, spikes in the middle of a finetuning session, we find that this gain is not consistent across all episodes. From the curve, it can be noted that at around 300 iterations, the loss reaches a local minimum while the accuracy gain increases. Although the accuracy gain continues to increase, it is unsteady (as shown in the accuracy at iterations 400 and 500). Overall, to observe a steady gain for different episodes, the model has to be trained longer to reach the global minimum. At the global minimum, the accuracy for a certain episode is not the best that has been reached; however, this accuracy is immune to episode change.

**Figure 3:**
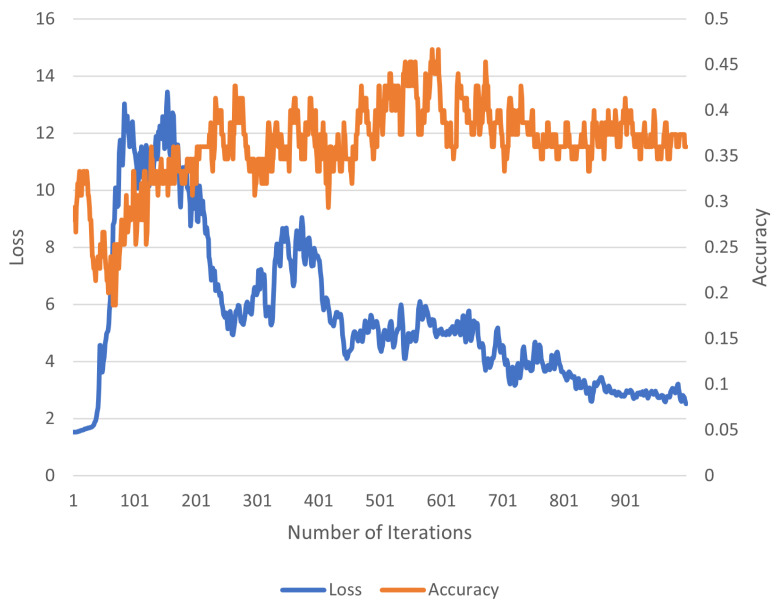
**Loss and accuracy for a different ISIC episodic batch than in Figure 2 over 1000 finetuning iterations.** This episode is different than the episode of Figure 2. Although the loss values are different, the same general trend is still observed.

Finally, we ablate three properties of FSS in Table 4. For our ablation experiments, we test FSS using the ISIC dataset (due to the challenging nature of its content) in a 5-way 5-shot manner. We examine the on-line finetuning duration, backbone selection, and on-line finetuning learning rate. Overall, we observe that longer finetuning sessions with lower learning rates enable effective improvements in terms of reconstruction and in terms of few-shot accuracy (as explored in Figure 2 and Figure 3). The two figures show that the finetuning session has to be long enough for the accuracy to be steady. Additionally, we discover that backbone selection is another critical component in FSS. Smaller backbones (such as ViT-Base) exhibit reduced finetuning performance vs. larger backbones (such as ViT-Large or ViT-Huge).

## 5. Discussion

This paper proposes FSS, a novel, fully self-supervised out-of-domain few-shot learning technique. FSS uses a masked autoencoder to adapt a vision transformer to new domains in an online fashion without using any labels. Hence, FSS is able to generalize to out-of-domain classes. To the best of our knowledge, this is the first attempt at self-supervised few-shot learning without additional supervised downstream training. The conducted experiments show the effectiveness of the proposed FSS, especially in out-of-domain performance. Our results show that FSS improves the performance of the vision transformer by gains of 1.05% , 0.12% , and 1.28% on the ISIC, EuroSat, and BCCD datasets, respectively.

Although our method has many advantages, especially in out-of-domain performance, we point out that there are several limitations. First, our method requires a ViT to function properly since, for the time being, there is no competitive masked autoencoder implemented in CNN. Second, our model converges slowly compared to metric-based methods.

To address the limitations of our method, in future work, we can explore more CNN-based methods for masked image modelling for our specific case. Moreover, we may integrate a generalized self-supervised contrastive learning approach into our method, leading to faster convergence.

## Figures and Tables

**Table 1:** Hyperparameters were used in our experiments.

Hyperparameter	Value
Online finetuning	
Learning rate	1 × $10^{-5}$
Epochs	1000
# of query images per episode	15
MAE Backbone (ViT Large)	
Image size	224
Masking %	75
Patch size	16
# of encoder layers	24
# of decoder layers	8
# of attention heads	16

**Table 2:** Out-of-domain 5-Way 5-Shot Results for ISIC and EuroSAT.

Method (Backbone)	Out-of-Domain	
	**ISIC**	**EuroSAT**
	**5w5s**	**5w5s**
ProtoNet (RN10)	39.57	73.29
RelationNet (RN10)	39.41	61.31
MetaOptNet (RN10)	36.28	64.44
CHEF (RN10)	41.26	74.15
FSS (ViT-large)	42.31	74.27
w/o FSS (ViT-large)	37.66	73.06

**Table 3:** Out-of-domain 5-Way 5-Shot Results for BCCD WBC (BCCD) with FSS. Results for previous approaches are sourced from previous work [50].

Method (Backbone)	Out-of-Domain
	**BCCD**
	**5w5s**
AmdimNet (Amdim)	48.35
EPNet + SSL (WRN28-10)	47.39
SimpleCNAPS (ResNet-18)	47.06
ProtoNet (CONV4)	46.89
S2M2R (WRN28-10)	44.15
PT + MAP (WRN28-10)	42.94
MAML (CONV4)	42.81
LaplacianShot (WRN28-10)	34.75
FSS (ViT-Large)	49.63
w/o FSS (ViT-Large)	48.08

**Table 4:** FSS Ablation studies using the ISIC dataset in 5-way 5-shot settings. For all non-encoder related tests, a ViT-Large encoder is used with ImageNet-1k pretrained weights. Significant results are in bold.

**(a)** On-line Finetuning Iterations: We find that longer finetuning sessions generally provide better results than shorter sessions.			**(b)** FSS Backbone Evaluation: We evaluate three different encoder designs (with ImageNet-1k pretrained weights) for FSS. To this end, we observe that ViT-Large provides strong performance for on-line, self-supervised finetuning.			**(c)** On-line Finetuning Learning Rate Evaluation: Four different learning rates are explored for on-line finetuning. We find that a lower learning rate is generally better; however, reducing too much leads to lack of improvement in reconstruction performance and accuracy.	
Iters.	Accuracy		Encoders	Accuracy		Learning Rate	Accuracy
50	37.82		ViT-Base	31.73		1 × $10^{-3}$	28.57
500	40.11		VIT-Large	42.31		1 × $10^{-4}$	40.93
1000	42.31		ViT-Huge	40.26		1 × $10^{-5}$	42.43
2000	39.37					1 × $10^{-6}$	37.48

## Data Availability

Not applicable.

## Abbreviation

The following abbreviation is used in this manuscript:

FSS	Few-shot Self-supervised

## References

[1] Bateni P., Goyal R., Masrani V., Wood F., Sigal L. Improved few-shot visual classification. Proceedings of the IEEE/CVF Conference on Computer Vision and Pattern Recognition.

[2] Hu S.X., Li D., Stühmer J., Kim M., Hospedales T.M. Pushing the limits of simple pipelines for few-shot learning: External data and fine-tuning make a difference. Proceedings of the IEEE/CVF Conference on Computer Vision and Pattern Recognition.

[3] Rodríguez P., Laradji I., Drouin A., Lacoste A. Embedding propagation: Smoother manifold for few-shot classification. Proceedings of the Computer Vision—ECCV 2020: 16th European Conference.

[4] Snell J., Swersky K., Zemel R. (2017). Prototypical networks for few-shot learning. Adv. Neural Inf. Process. Syst..

[5] Munkhdalai T., Yuan X., Mehri S., Trischler A. Rapid adaptation with conditionally shifted neurons. Proceedings of the International Conference on Machine Learning.

[6] Rusu A.A., Rao D., Sygnowski J., Vinyals O., Pascanu R., Osindero S., Hadsell R. (2018). Meta-learning with latent embedding optimization. arXiv.

[7] Antoniou A., Storkey A.J. (2019). Learning to learn by self-critique. Adv. Neural Inf. Process. Syst..

[8] Finn C., Abbeel P., Levine S. Model-agnostic meta-learning for fast adaptation of deep networks. Proceedings of the International Conference on Machine Learning.

[9] Chen D., Chen Y., Li Y., Mao F., He Y., Xue H. Self-supervised learning for few-shot image classification. Proceedings of the ICASSP 2021–2021 IEEE International Conference on Acoustics, Speech and Signal Processing (ICASSP).

[10] Osman I., Shehata M.S. (2022). Few-Shot Learning Network for Out-of-Distribution Image Classification. IEEE Trans. Artif. Intell..

[11] Vinyals O., Blundell C., Lillicrap T., Wierstra D., Kavukcuoglu K. Matching networks for one shot learning. Proceedings of the Advances in Neural Information Processing Systems.

[12] Laenen S., Bertinetto L. (2021). On Episodes, Prototypical Networks, and Few-Shot Learning. Adv. Neural Inf. Process. Syst..

[13] Ravi S., Larochelle H. Optimization as a Model for Few-Shot Learning. Proceedings of the International Conference on Learning Representations.

[14] Hospedales T., Antoniou A., Micaelli P., Storkey A. (2022). Meta-Learning in Neural Networks: A Survey. IEEE Trans. Pattern Anal. Mach. Intell..

[15] Finn C., Rajeswaran A., Kakade S., Levine S. Online Meta-Learning. Proceedings of the 36th International Conference on Machine Learning.

[16] Li Z., Zhou F., Chen F., Li H. (1707). Meta-SGD: Learning to Learn Quickly for Few Shot Learning. arxiv.

[17] Antoniou A., Edwards H., Storkey A. How to train your MAML. Proceedings of the International Conference on Learning Representations.

[18] Franceschi L., Donini M., Frasconi P., Pontil M. Forward and Reverse Gradient-Based Hyperparameter Optimization. Proceedings of the 34th International Conference on Machine Learning.

[19] Li Y., Yang Y., Zhou W., Hospedales T. Feature-Critic Networks for Heterogeneous Domain Generalisation. Proceedings of the Thirty-Sixth International Conference on Machine Learning.

[20] Wichrowska O., Maheswaranathan N., Hoffman M.W., Colmenarejo S.G., Denil M., de Freitas N., Sohl-Dickstein J. Learned Optimizers That Scale and Generalize. Proceedings of the 34th International Conference on Machine Learning—Volume 70.

[21] Baydin A.G., Cornish R., Rubio D.M., Schmidt M., Wood F. Online Learning Rate Adaptation with Hypergradient Descent. Proceedings of the International Conference on Learning Representations.

[22] Meier F., Kappler D., Schaal S. Online Learning of a Memory for Learning Rates. Proceedings of the IEEE International Conference on Robotics and Automation (ICRA).

[23] Kingma D.P., Welling M. Auto-Encoding Variational Bayes. Proceedings of the 2nd International Conference on Learning Representations, ICLR 2014.

[24] Goodfellow I., Pouget-Abadie J., Mirza M., Xu B., Warde-Farley D., Ozair S., Courville A., Bengio Y. (2020). Generative adversarial networks. Commun. Acm.

[25] Ho J., Jain A., Abbeel P. (2020). Denoising diffusion probabilistic models. Adv. Neural Inf. Process. Syst..

[26] Klys J., Snell J., Zemel R. (2018). Learning latent subspaces in variational autoencoders. Adv. Neural Inf. Process. Syst..

[27] Schonfeld E., Ebrahimi S., Sinha S., Darrell T., Akata Z. Generalized Zero- and Few-Shot Learning via Aligned Variational Autoencoders. Proceedings of the IEEE/CVF Conference on Computer Vision and Pattern Recognition (CVPR).

[28] Zhang R., Che T., Ghahramani Z., Bengio Y., Song Y. (2018). Metagan: An adversarial approach to few-shot learning. Adv. Neural Inf. Process. Syst..

[29] Yang S., Liu L., Xu M. Free Lunch for Few-shot Learning: Distribution Calibration. Proceedings of the International Conference on Learning Representations.

[30] Radford A., Narasimhan K., Salimans T., Sutskever I. (2018). Improving Language Understanding by Generative Pre-Training.

[31] Brown T., Mann B., Ryder N., Subbiah M., Kaplan J.D., Dhariwal P., Neelakantan A., Shyam P., Sastry G., Askell A. (2020). Language models are few-shot learners. Adv. Neural Inf. Process. Syst..

[32] Radford A., Wu J., Child R., Luan D., Amodei D., Sutskever I. (2019). Language models are unsupervised multitask learners. Openai Blog.

[33] Devlin J., Chang M.W., Lee K., Toutanova K. (2018). Bert: Pre-training of deep bidirectional transformers for language understanding. arXiv.

[34] LeCun Y., Misra I. Self-Supervised Learning: The Dark Matter of Intelligence. https://ai.meta.com/blog/self-supervised-learning-the-dark-matter-of-intelligence/.

[35] Balestriero R., Ibrahim M., Sobal V., Morcos A., Shekhar S., Goldstein T., Bordes F., Bardes A., Mialon G., Tian Y. (2023). A Cookbook of Self-Supervised Learning. arXiv.

[36] Bromley J., Guyon I., LeCun Y., Säckinger E., Shah R., Cowan J., Tesauro G., Alspector J. (1993). Signature Verification using a “Siamese” Time Delay Neural Network. Advances in Neural Information Processing Systems.

[37] Schroff F., Kalenichenko D., Philbin J. FaceNet: A Unified Embedding for Face Recognition and Clustering. Proceedings of the IEEE Conference on Computer Vision and Pattern Recognition (CVPR).

[38] Wang W., Arora R., Livescu K., Bilmes J. On Deep Multi-View Representation Learning. Proceedings of the 32nd International Conference on International Conference on Machine Learning—Volume 37.

[39] Andrew G., Arora R., Bilmes J., Livescu K. Deep Canonical Correlation Analysis. Proceedings of the 30th International Conference on Machine Learning.

[40] Zbontar J., Jing L., Misra I., LeCun Y., Deny S. Barlow twins: Self-supervised learning via redundancy reduction. Proceedings of the International Conference on Machine Learning.

[41] Walsh R., Osman I., Shehata M. (2023). Masked Embedding Modeling With Rapid Domain Adjustment for Few-Shot Image Classification. IEEE Trans. Image Process..

[42] Grill J.B., Strub F., Altché F., Tallec C., Richemond P., Buchatskaya E., Doersch C., Avila Pires B., Guo Z., Gheshlaghi Azar M., Larochelle H., Ranzato M., Hadsell R., Balcan M., Lin H. (2020). Bootstrap Your Own Latent—A New Approach to Self-Supervised Learning. Advances in Neural Information Processing Systems.

[43] Caron M., Touvron H., Misra I., Jégou H., Mairal J., Bojanowski P., Joulin A. Emerging Properties in Self-Supervised Vision Transformers. Proceedings of the IEEE/CVF International Conference on Computer Vision (ICCV).

[44] He K., Chen X., Xie S., Li Y., Dollár P., Girshick R. Masked Autoencoders Are Scalable Vision Learners. Proceedings of the IEEE/CVF Conference on Computer Vision and Pattern Recognition (CVPR).

[45] Dosovitskiy A., Beyer L., Kolesnikov A., Weissenborn D., Zhai X., Unterthiner T., Dehghani M., Minderer M., Heigold G., Gelly S. An Image is Worth 16x16 Words: Transformers for Image Recognition at Scale. Proceedings of the International Conference on Learning Representations.

[46] Helber P., Bischke B., Dengel A., Borth D. (2019). Eurosat: A novel dataset and deep learning benchmark for land use and land cover classification. IEEE J. Sel. Top. Appl. Earth Obs. Remote Sens..

[47] Codella N., Rotemberg V., Tschandl P., Celebi M.E., Dusza S., Gutman D., Helba B., Kalloo A., Liopyris K., Marchetti M. (2019). Skin lesion analysis toward melanoma detection 2018: A challenge hosted by the international skin imaging collaboration (isic). arXiv.

[48] Shenggan (2022). BCCD Dataset. https://github.com/Shenggan/BCCD_Dataset.

[49] Mangla P., Kumari N., Sinha A., Singh M., Krishnamurthy B., Balasubramanian V.N. Charting the Right Manifold: Manifold Mixup for Few-shot Learning. Proceedings of the IEEE/CVF Winter Conference on Applications of Computer Vision (WACV).

[50] Walsh R., Abdelpakey M.H., Shehata M.S., Mohamed M.M. (2022). Automated human cell classification in sparse datasets using few-shot learning. Sci. Rep..

